# MicroRNA in cancer therapy: breakthroughs and challenges in early clinical applications

**DOI:** 10.1186/s13046-025-03391-x

**Published:** 2025-04-21

**Authors:** Maria Teresa Di Martino, Pierosandro Tagliaferri, Pierfrancesco Tassone

**Affiliations:** Department of Experimental and Clinical Medicine, Magna Graecia University, Catanzaro, Italy

**Keywords:** microRNA, miRNA, miRNA inhibitor, Non-coding RNA, ncRNA, Phase 1, miRNA therapeutics, First-in-human, Early clinical trials, Clinical trials, Locked nucleic acid, LNA

## Abstract

MicroRNAs (miRNAs) have emerged as pivotal regulators in cancer biology, influencing tumorigenesis, progression, and resistance to therapy. Their ability to modulate multiple oncogenic and tumor-suppressive pathways positions them as promising therapeutic tools or targets. This review examines the dual role of miRNAs in solid and hematological malignancies, starting from their dysregulation in various cancer types. Therapeutic approaches, including miRNA replacement and inhibition strategies, are discussed alongside innovative delivery systems such as lipid nanoparticles and exosomes. Despite their transformative potential, challenges persist, including off-target effects, immune activation, and delivery inefficiencies. Recent clinical trials demonstrate both progress and hurdles, underscoring the need for advanced strategies to optimize specificity and minimize toxicity. This review provides an updated comprehensive overview of the current landscape of miRNA-based therapies under early clinical investigation and explores future directions for integrating these approaches into precision oncology.

## Introduction

Cancer continues to pose a major global health challenge, with nearly 19 million new diagnoses and around 10 million fatalities annually [1, 2]. Despite notable progress in targeted therapies and immunotherapies, which have significantly improved survival in several patients, their impact is constrained by challenges such as treatment resistance and disease recurrence or progression. These events limit the chance of disease control by currently available therapeutics, emphasizing the need of innovative, personalized strategies. While targeted therapies and immunotherapies offer enhanced precision by focusing on specific molecular targets or leveraging the immune system, unfortunately, their success is not universal. This is largely due to cancer’s complexity and heterogeneity, which result in differential sensitivity among patients, as well as high costs and logistic hurdles associated with these treatments, making widespread accessibility difficult.

RNA-based therapies, particularly those involving non-coding RNAs (ncRNAs), like microRNAs (miRNAs), represent a new emerging frontier, a promising alternative to address these challenges [3]. MiRNAs are the most studied class of regulatory ncRNAs of approximately 20–24 nucleotides, which regulate gene expression by binding to complementary sequences on messenger RNAs (mRNAs), leading to mRNA degradation or translation inhibition. Through this post-transcriptional regulation, miRNAs influence critical biological processes, such as cell proliferation, differentiation, apoptosis, and metabolism, thereby helping maintain cellular homeostasis. Dysregulation of miRNAs can disrupt these processes, contributing to the development of diseases, including cancer, by either promoting oncogenic pathways or hampering tumor-suppressive functions [4, 5].

MiRNA-based therapies exploit the dual role of miRNAs in cancer, targeting either oncogenic miRNAs (oncomiRs) or tumor-suppressive miRNAs to restore balance. Oncogenic miRNAs, when overexpressed, can drive tumor progression, whereas downregulated tumor-suppressive miRNAs fail to inhibit malignant growth. Therapeutic strategies include the use of miRNA mimics to restore the function of downregulated tumor-suppressive miRNAs or miRNA inhibitors (antimiRs) to neutralize overexpressed oncomiRs. Given their small size and chemical properties, miRNAs are conducive to synthesis and can be packaged into delivery systems tailored to target tumor cells specifically. This targeted approach could reduce the off-target effects seen in conventional treatments like chemotherapy and radiation, potentially minimizing damage to healthy cells [6, 7].

As research advances, miRNA-based therapies hold potential to enrich oncology with personalized treatment options tailored to the unique miRNA profiles of individual tumors. This approach could not only enhance therapeutic efficacy but also mitigate adverse effects, thus leading to better outcomes for patients battling this complex disease [8, 9].

## MiRNA dysregulation in cancer

The dysregulation of miRNAs in solid tumors and hematological malignancies highlights the complex role that these molecules play in cancer development and progression [10, 11].

Some miRNAs, such as miR-21 and miR-221, are frequently overexpressed in various cancers and promote tumorigenesis by targeting key tumor suppressor genes. For instance, miR-21, which is upregulated in breast, lung, gastric, and brain cancers, inhibits tumor suppressors like PTEN, PDCD4, and TPM1, thereby enhancing tumor growth, metastasis, and chemoresistance [12]. Similarly, miR-221 is highly expressed in hepatocellular carcinoma, melanoma, colon cancer and renal cell carcinoma, where it downregulates cell cycle inhibitors like p27 and p57 to promote uncontrolled proliferation [13–16]. Other upregulated miRNAs, including miR-155, miR-182, and miR-200b, exhibit tumor-specific expression patterns, contributing to processes such as cell proliferation, cell survival, invasion, and metastasis.

Conversely, the downregulation of tumor suppressor miRNAs removes their inhibitory effects on oncogenic pathways, further driving tumor progression. For example, miR-143 and miR-145 are downregulated in bladder, breast, lung, colorectal, and pancreatic cancers [17, 18], while the Let-7 family is suppressed in lung cancer, where its loss leads to the unchecked activity of oncogenes such as RAS and HMGA2 [19]. Similarly, miR-126 and miR-125b are reduced in breast, lung, and gastric cancers, underscoring their importance in apoptosis and cellular differentiation [20, 21]. Finally, the miR-34 family is a tumor-suppressive miRNA in diverse cancer types, raising high interest in a broad-spectrum therapeutic approach [22]. This dual role of miRNAs highlights their complexity, as miR-21 and miR-221, while largely oncogenic, may also participate in immune regulation or stress responses depending on the tumor context. The tumor-specific dysregulation patterns of miRNAs not only demonstrate their diverse biological roles but also emphasize their potential as diagnostic, prognostic, and therapeutic biomarkers. A detailed summary of the key miRNAs dysregulated across solid tumors is provided in Table 1, illustrating the overlap and tumor-specific expression patterns of these molecules.

**Table 1:** Expression patterns of key MiRNAs in solid tumors

Solid Tumor	Upregulated miRNAs/Oncogene miRNAs	Downregulated miRNAs/Tumor Suppressor miRNAs
Bladder Cancer	miR-21, miR-182, miR-200a, miR-23b, miR-1274a	miR-125b, miR-145, miR-200c, miR-143, miR-218, miR-497, miR-665
Brain Cancer	miR-17/92, miR-21, miR-221, miR-222	miR-7, miR-34a, miR-124, miR-128, miR-136, miR-153, miR-137, miR-199b, miR-324-5p, miR-326, miR-451
Breast Cancer	miR-10b, miR-21, miR-155, miR-210, miR-10b, miR-27a, miR-141, miR-766	miR-31, miR-125b, miR-205, let-7, miR-7, miR-30, miR-34a, miR-125, miR-127, miR-140, miR-143
Cervical Cancer	miR-10a, miR-21, miR-221	miR-34a, miR-143, miR-145
Colorectal Cancer	miR-17-5p, miR-21, miR-155, miR-126, miR-145, miR-155-5p, miR-592, miR-1274a	miR-34a, miR-143, miR-145, miR-137-3p, miR-3622a
Esophageal Cancer	miR-21, miR-25, miR-155	miR-100, miR-143, miR-145
Gastric Cancer	miR-21, miR-106b, miR-221, miR-21, miR-25, miR-27a, miR-93, miR-106b, miR-155	miR-101, miR-126, miR-218, let-7, miR-15b, miR-16, miR-122a, miR-141, miR-143, miR-876
Hepatocellular Carcinoma	miR-21, miR-221, miR-222, miR-224, miR-21, miR-25, miR-27a, miR-93	miR-122, miR-125b, miR-199a, let-7, miR-15b, miR-16, miR-141, miR-451
Head and Neck Cancer	miR-106b, miR-142-3p, miR-423, miR-6728, miR-99a, miR-20a, miR-19b	miR-375, miR-125b, let-7a
Lung Cancer	miR-21, miR-31, miR-155, miR-210, miR-17-92, miR-21, miR-31, miR-518b, miR-629	miR-34a, miR-126, miR-200b, let-7, miR-1, miR-124a, miR-125a, miR-183, miR-190b
Melanoma	miR-21, miR-182, miR-221, miR-21, miR-221, miR-222	miR-34b, miR-137, miR-211, miR-7, miR-493
Ovarian Cancer	miR-21, miR-200a, miR-200b, let-7, miR-199a, miR-223	miR-100, miR-125b, miR-145, miR-126, miR-134, miR-377
Pancreatic Cancer	miR-21, miR-155, miR-210, miR-200b	miR-34a, miR-101, miR-124, miR-142
Prostate Cancer	miR-21, miR-221, miR-222, miR-141	miR-34a, miR-125b, miR-145, let-7c, miR-34, miR-125b
Renal Cell Carcinoma	miR-21, miR-210, miR-221, miR-19, miR-25, miR-1274a, miR-142, miR-154 miR-543	miR-106a, miR-199a, miR-363, miR-384, miR-622
Thyroid Cancer	miR-146b, miR-221, miR-222	miR-1, miR-138, miR-206

Similarly, the dysregulation of miRNAs in hematological malignancies significantly contributes to cancer onset, progression, and resistance to therapy, reflecting their diverse roles as oncogenes or tumor suppressors [23]. In multiple myeloma (MM), for example, miR-21 and miR-221 are frequently upregulated, enhancing cell survival, proliferation, and drug resistance [24, 25], while tumor suppressive miRNAs like miR-29b and miR-34a are downregulated [26, 27], contributing to disease aggressiveness and poor prognosis. Similarly, in acute lymphoblastic leukemia (ALL), upregulated miRNAs such as miR-21, miR-221, miR-155, and miR-181a promote leukemogenesis by enhancing cell proliferation and shortening survival [28–31], whereas the downregulation of miR-34a, miR-29b, and miR-223 removes critical checkpoints on cell cycle regulation and apoptosis, enabling malignant growth [29]. Moreover, it has been shown that miRNA expression signatures are associated with cytogenetics and prognosis in acute myeloid leukemia (AML) and some miRNA expression patterns correlate with survival [32]. Notably, miR-21 emerges as a common oncogenic factor across various hematological malignancies, including chronic lymphocytic leukemia (CLL), and diffuse large B-cell lymphoma (DLBCL), underscoring its potential as both a biomarker and therapeutic target [33]. In DLBCL and Hodgkin lymphoma (HL), upregulated miR-17, miR-155, and miR-210 contribute to tumor progression, while downregulated miR-34a, miR-144, and miR-150 highlight their tumor suppressive roles [34]. Chronic myeloid leukemia (CML) further exemplifies this dual nature, with miR-21 and miR-155 acting as oncogenes, along with the frequent loss of tumor suppressor miRNAs like miR-146 and miR-150, demonstrate common pathways that drive hematological malignancies [35]. These findings emphasize the therapeutic potential of targeting specific miRNAs to control leukemogenesis and lymphomagenesis. Table 2 summarizes the expression patterns of key miRNAs in hematological malignancies, offering a comprehensive view of their oncogenic and tumor suppressive roles.

**Table 2:** Expression patterns of key MiRNAs in hematological malignancies

Hematopoietic Malignancy	Upregulated miRNAs	Downregulated miRNAs
Acute Lymphoblastic Leukemia (ALL)	miR-21, miR-155, miR-181a	miR-34a, miR-126, miR-150
Acute Myeloid Leukemia (AML)	miR-10b, miR-21, miR-155, miR-181a	miR-34a, miR-29b, miR-223
Chronic Lymphocytic Leukemia (CLL)	miR-21, miR-155	miR-15a, miR-16-1, miR-34a
Chronic Myeloid Leukemia (CML)	miR-21, miR-181b	miR-30a, miR-223
Diffuse Large B-Cell Lymphoma (DLBCL)	miR-17, miR-21, miR-155, miR-222	miR-34a, miR-144, miR-181a
Hodgkin Lymphoma (HL)	miR-21, miR-155, miR-210	miR-26a, miR-125b, miR-150
Multiple Myeloma (MM)	miR-21, miR-221, miR-222, miR-223	miR-29b, miR-34a, miR-125b, miR-155

Despite the association of upregulated miRNAs with oncogenic activity, not all upregulated miRNAs act as oncogenes. Their function and classification can depend heavily on cellular context and target interactions, which may vary among cancer types. Some miRNAs exhibit dual roles, acting as both oncogenes and tumor suppressors depending on the tumor microenvironment and specific signaling pathways involved [36]. Recognizing the nuanced role of miRNAs in cancer can aid in developing targeted miRNA-based therapies for a wide array of malignancies.

### MiRNA replacement and Inhibition strategies

miRNA-based therapeutic strategies, including miRNA replacement and inhibition, are emerging as promising approaches for cancer treatment, offering the potential to restore or suppress the function of specific miRNAs that are dysregulated in various solid and hematological malignancies [37–40]. miRNA mimics, which are synthetic versions of natural miRNAs, are designed to replace the lost tumor-suppressive functions of these miRNAs. These mimics are introduced into cancer cells to restore the activity of miRNAs like let-7 [41], miR-34a [27, 42, 43], or miR-29b [26, 44–49] which are often downregulated in malignancies. By reactivating these tumor-suppressive miRNAs, miRNA mimics can suppress tumor growth, induce apoptosis, and inhibit metastasis. Many preclinical studies have demonstrated the efficacy of miRNA mimics in reducing tumor growth and improving the response to conventional therapies, making them attractive candidates for therapeutic development.

**Fig. 1:**
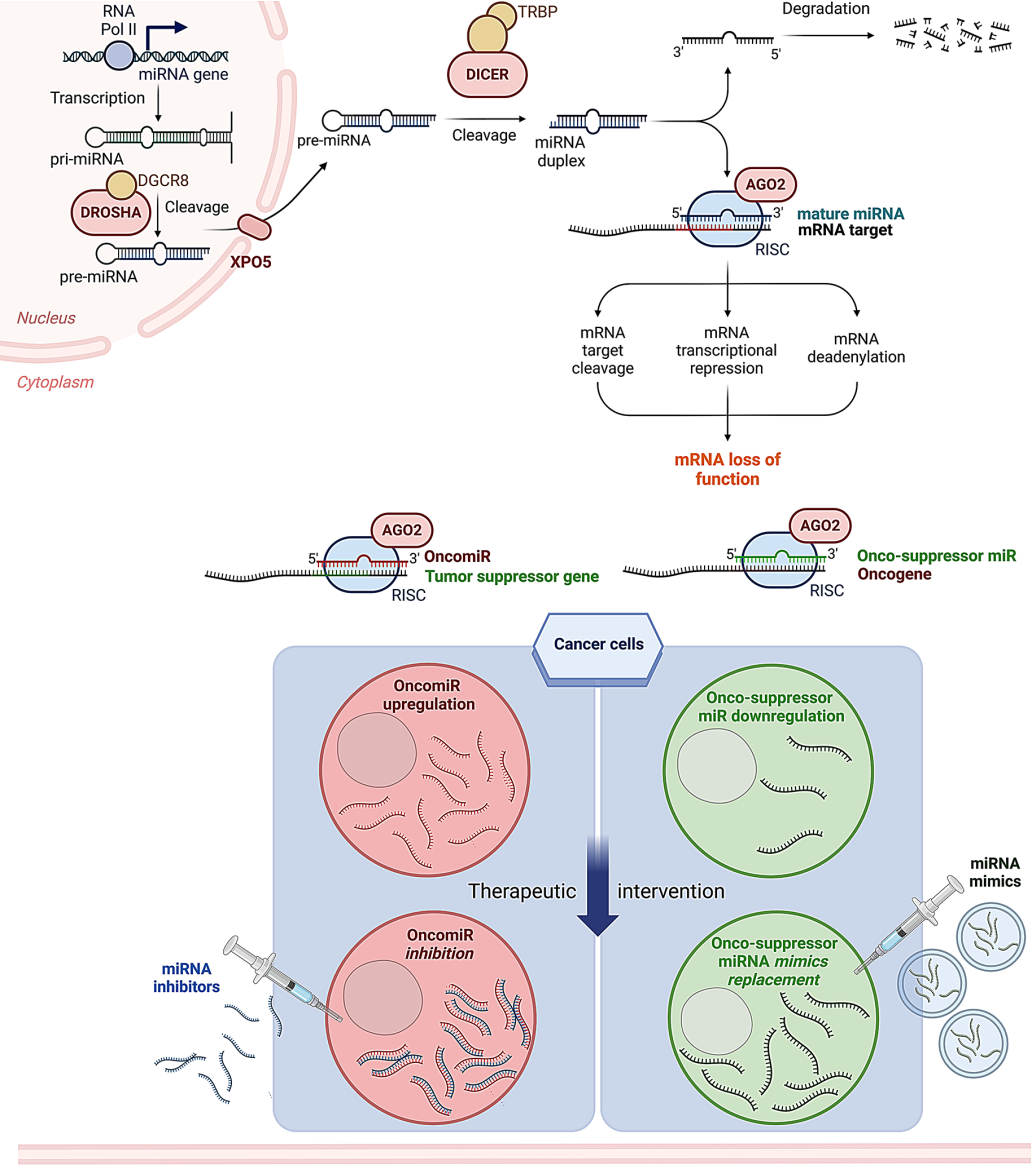
miRNA Biogenesis and Its Potential as a Therapeutic Target in Cancer. miRNAs biogenesis is a multi-step process that begins in the nucleus where are transcribed as pri-miRNAs by RNA polymerase II. Then the pri-miRNAs are cleaved by the Drosha-DGCR8 complex into precursor pre-miRNAs that leave the cytoplasm by Exportin-5 where the enzyme Dicer further processes pre-miRNAs into mature miRNAs. The mature miRNAs are incorporated into the RNA-induced silencing complex (RISC), where they guide degradation or repression of target mRNAs, ultimately affecting protein production. miRNAs regulate genes involved in cell growth, apoptosis, and differentiation, their dysregulation is often linked to cancer. They can act as either: OncomiRs (Oncogenic miRNAs)– Promote cancer by suppressing tumor suppressor genes, or Tumor Suppressor miRNAs– Prevent cancer by inhibiting oncogenes. This makes miRNAs promising therapeutic targets in cancer by miRNA inhibition– blocking oncomiRs with ASOs or miRNA mimics– restoring tumor suppressor miRNAs to suppress cancer growth

On the other hand, miRNA inhibitors (also called antimiRs) are antisense oligonucleotides designed to block the activity of oncogenic miRNAs that are upregulated in cancers. These inhibitors are typically intended to bind the target miRNA and prevent it from interacting with its mRNA targets, thus reversing the oncogenic effects of miRNAs. By targeting and inhibiting specific oncomiRs, such as miR-21 [24, 50–52], miR-221 [14, 25, 53–57], and miR-17/92 cluster [58], these inhibitors can restore the activity of tumor suppressor pathways, reduce cell proliferation, and also enhance the sensitivity of cancer cells to chemotherapy [59, 60]. Figure 1 illustrates the biogenesis pathway of miRNAs, the effects of their dysregulation in tumor cells, and their potential as targets for miRNA-based therapeutic approaches.

Both miRNA mimics and inhibitors face challenges related to delivery mechanisms, stability in the bloodstream, and off-target effects. To overcome these barriers, innovative delivery systems, such as nanoparticles and liposomes, are being developed to efficiently deliver miRNA-based drugs to the tumor site while minimizing systemic toxicity. Furthermore, understanding the context-dependent functions of miRNAs is crucial for optimizing their therapeutic application. Therefore, the ability to precisely target specific miRNA pathways in distinct cancer contexts will be critical for the success of miRNA-based therapies.

Therefore, miRNA replacement and inhibition strategies have the potential to expand the landscape of cancer therapy by offering a selectively targeted approach to modulate the expression of key regulators of tumorigenesis. The development of miRNA mimics and inhibitors as therapeutic agents has shown promising results in preclinical and early clinical trials, and ongoing research is focused on refining delivery systems and improving specificity to ensure their clinical success in treating cancer.

### Delivery systems and stability challenges of miRNA-based therapies

The successful development of miRNA-based therapeutics for cancer treatment relies on addressing critical challenges, including the instability of miRNAs in circulation and their limited ability to penetrate target cells. Due to their susceptibility to rapid degradation by RNases and their hydrophilic, negatively charged nature, effective delivery systems are essential to enhance miRNA stability, bioavailability and specificity.

Among the most promising solutions for delivery are lipid nanoparticles (LNPs), polymeric nanoparticles, and exosomes. LNPs shield miRNAs from enzymatic degradation, enable controlled release, and facilitate cellular uptake. They can be further modified with targeting ligands, such as antibodies or peptides, to improve delivery precision, thereby minimizing off-target effects and enhancing therapeutic outcomes. LNPs have shown considerable success in RNA-based therapies, including siRNA and mRNA treatments, and are now being explored extensively for miRNA delivery in cancer models [61]. Similarly, polymeric nanoparticles, particularly those made from biocompatible materials like PLGA (poly(lactic-co-glycolic acid)), offer tumor-specific delivery by responding to stimuli such as pH shifts or enzymatic activity within the tumor microenvironment [62]. This enables controlled, localized miRNA release, which enhances therapeutic efficacy. Exosome-based delivery systems have also gained traction due to the natural role of exosomes in intercellular communication [63]. These vesicles efficiently transport miRNAs without triggering immune responses, offering superior biocompatibility compared to synthetic nanoparticles. Additionally, exosomes can be engineered to carry higher miRNA payloads and deliver them selectively to target cells. However, challenges remain in the large-scale production and clinical translation of exosome-based therapies [64].

For improved stability, high affinity and specificity of binding, Locked Nucleic Acids (LNAs) represent a promising approach. LNAs are chemically modified RNA analogs with a rigid ribose structure that confers resistance to nuclease degradation while significantly enhancing affinity for target miRNAs. LNAs have demonstrated success in inhibiting oncogenic miRNAs, such as miR-21 [24] and miR-221 [56], as well as the inhibition mediated by LNA-miR-361 [65]. LNA-miR-29b [66] and LNA-miR-34a [67] demonstrated efficient tumor cell growth modulation in vitro and in vivo. By silencing oncomiRs, LNAs can restore tumor suppressor gene function, inhibit tumor progression, and provide prolonged therapeutic effects, reducing the need for frequent dosing and improving patient compliance [68–70].

Other innovative tools include CRISPR-Cas9-based systems, which allow for precise manipulation of miRNA expression directly at the genomic level. These interventions enable durable and highly specific regulation of miRNA activity, holding significant potential for treating cancers driven by miRNA dysregulation [71]. Furthermore, miRNA sponges, engineered constructs that sequester specific miRNAs, have emerged as tools to inhibit harmful miRNAs that contribute to tumor growth [72]. Delivery of these constructs using nanoparticles or liposomes ensures stability and efficient intracellular uptake. Lastly, small molecules capable of modulating miRNA expression are being explored as adjuncts, either enhancing tumor-suppressive miRNAs (e.g., miR-34a, let-7) or inhibiting oncogenic miRNAs.

Although viral vectors, such as adenoviruses and lentiviruses, are efficient tools for miRNA delivery due to their high gene transfer potential, they are challenging for eliciting immune responses and mutagenic risks. Ongoing advancements aim to optimize these vectors to balance their efficiency with safety [73].

In summary, continued innovation in delivery platforms, such as lipid nanoparticles, polymeric carriers, exosomes, LNAs, and genome-editing tools, remains crucial for overcoming the limitations of miRNA-based therapeutics. These technologies not only enhance the stability, targeting, and bioavailability of miRNAs but also minimize side effects, ultimately improving the efficacy and clinical translation of miRNA-based cancer therapies.

### Off-target effects and toxicity

miRNA-based therapies, including the use of miRNA mimics and miRNA inhibitors, offer significant promise for cancer treatment but hold concerns over off-target effects, immune responses, and unintended toxicity. Off-target effects, wherein miRNA mimics or inhibitors bind to and regulate unintended genes, represent a primary challenge. Taking into account that miRNAs can target multiple mRNA transcripts due to their partial complementarity, synthetic miRNAs may inadvertently affect various cellular pathways, potentially disrupting normal cell functions or even promoting oncogenesis in non-cancerous tissues [74, 75]. For example, miRNA mimics that restore tumor-suppressive functions could downregulate essential genes in healthy cells, while anti-miRs aimed at antagonizing oncogenic miRNAs may suppress non pro-tumors miRNAs with sequences similarity, leading to undesirable effects [76].

Another layer of complexity arises from the delivery systems used for miRNA therapeutics, which include, as above mentioned, lipid nanoparticles, polymeric carriers, and viral vectors. These systems may also introduce toxicity. Lipid nanoparticles, commonly used for miRNA delivery, can disrupt cell membranes and accumulate in organs, like the liver, causing hepatic toxicity and inflammation [77]. Viral vectors, while effective in delivering genetic material, pose risks such as insertional mutagenesis, which can lead to oncogenesis due to unintended integration into the host genome [78]. Furthermore, chemical modifications applied to enhance the stability of miRNAs, such as 2’-O-methyl or LNA modifications, can increase immune activation, as these alterations may trigger toll-like receptors (TLRs) on immune cells, leading to inflammatory responses and tissue damage [79].

Immune responses are another critical concern, as miRNA-based therapies can inadvertently activate both local and systemic immune reactions. Delivery systems, particularly lipid-based nanoparticles, can activate the complement system, leading to hypersensitivity reactions and, in severe cases, anaphylaxis, a phenomenon known as complement activation-related pseudoallergy (CARPA) [80]. Innate immune activation is also possible, as some RNA molecules are recognized as pathogenic by TLRs, resulting in the release of pro-inflammatory cytokines [81]. To mitigate these issues, researchers are investigating immune compliant strategies, such as polyethylene glycol (PEG) coating on nanoparticles, to reduce immunogenicity; however, these modifications can reduce delivery efficiency and may introduce additional challenges [82].

Adding to the complexity of this scenario is the fact that miRNAs can exhibit context-dependent functions, meaning that a miRNA functioning as a tumor suppressor in one cancer type may act as an oncogene in another. For example, while miR-21 is often upregulated in various cancers and is generally targeted as an oncomiR, it may play protective roles in other tissues or conditions. Introducing anti-miR-21 in such contexts could harm normal cells or even worsen certain conditions, emphasizing the need for thorough evaluation of each miRNA’s roles across different biological settings [7]. This context-dependency, combined with miRNAs’ broad regulatory functions, underscores the need for precise targeting and a deep understanding of each candidate’s effects to avoid adverse side effects and optimize therapeutic outcomes [83].

**Fig. 2:**
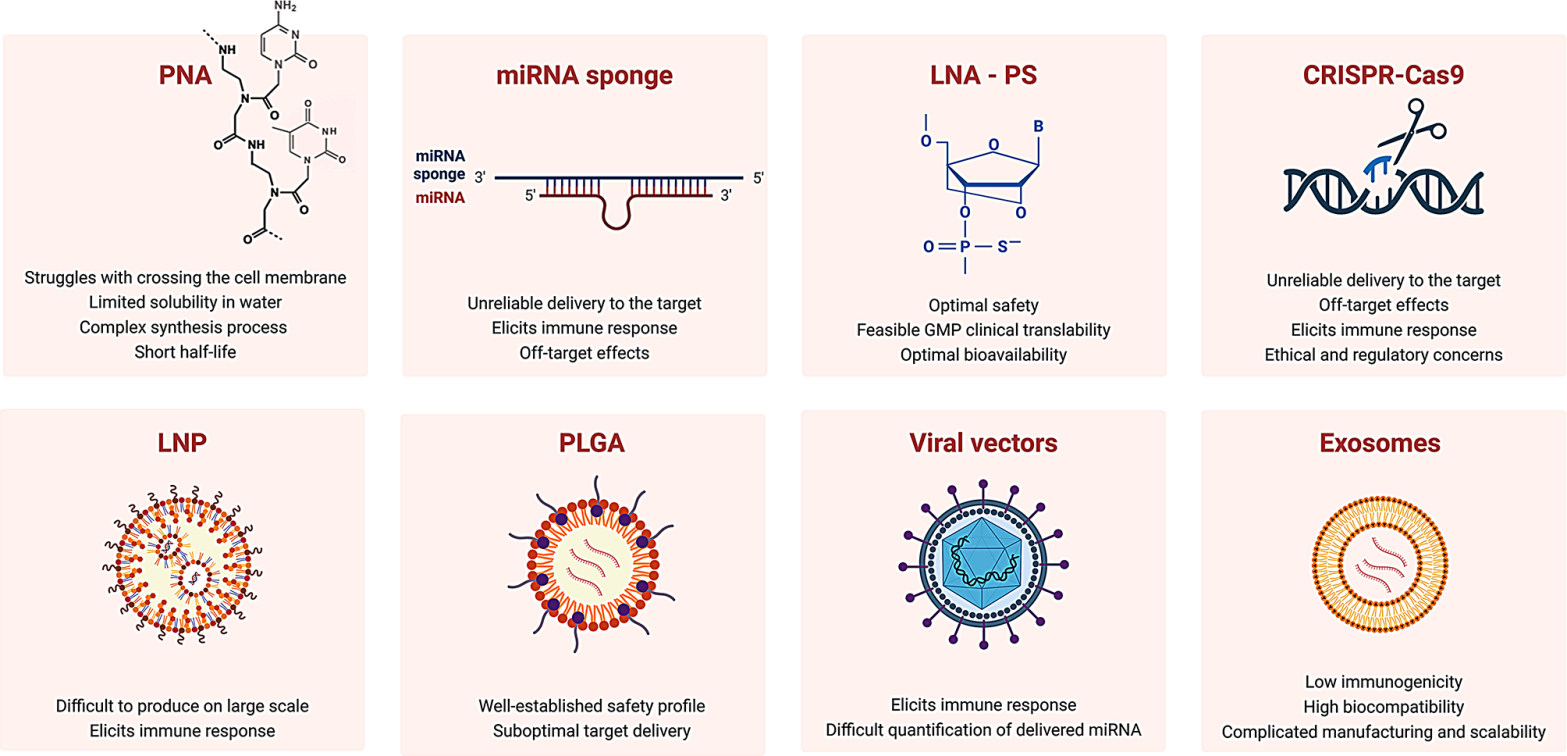
Strengths and Challenges in miRNA-Based Therapeutic Approaches. miRNA-based therapies show significant promise for cancer treatment, but various challenges must be addressed. Chemical modifications of ASOs or advanced delivery systems help overcome key issues, including: delivery efficiency, stability and degradation, off-target effects, immune response, scalability and manufacturing, regulatory and clinical validation

Overall, miRNA mimics and anti-miRNA inhibitors hold substantial therapeutic potential but must overcome significant hurdles related to off-target effects, toxicity, and immune response. Refining delivery systems, minimizing immune activation, and improving targeting specificity remain crucial areas of research to ensure the safe and effective clinical application of these therapies [77]. Figure 2 recapitulates the main challenges of miRNA-based therapeutics.

### Regulatory challenges and commercial hurdles

The development and commercialization of miRNA therapeutics face significant regulatory and manufacturing hurdles. One of the primary regulatory challenges for miRNA therapies, whether miRNA mimics or miRNA inhibitors, is meeting stringent quality control and safety standards required for clinical use. Given the complexity of miRNA molecules, regulatory bodies like the FDA and EMA require rigorous formal proofs on the safety, efficacy, and specificity before approval of miRNA-based treatments. A major concern is off-target effects, as miRNAs regulate multiple mRNAs, potentially leading to unintended interactions that could affect non-cancerous cells and contribute to toxicity [74, 84].

Furthermore, delivery systems used to ensure miRNA stability and targeting, such as lipid nanoparticles, viral vectors and conjugated oligonucleotide approaches, introduce additional regulatory scrutiny. These delivery platforms must demonstrate not only effective tissue and cellular targeting but also minimal immunogenicity and systemic toxicity. Regulatory agencies require comprehensive biodistribution, pharmacokinetic (PK), and pharmacodynamic (PD) studies to assess how miRNA therapeutics are absorbed, distributed, metabolized, and eliminated [85].The long-term persistence of miRNA drugs in non-target tissues is another safety concern that must be formally addressed in preclinical models according to Good Laboratory Practice (GLP) standards before moving to human trials.

Manufacturing problems further challenge the clinical translation of miRNA therapeutics. Unlike small-molecule, miRNAs are inherently unstable and susceptible to degradation, making batch-to-batch consistency and quality control a critical concern. Even minor modifications in the manufacturing process can impact the stability and efficacy of miRNA products. Regulatory authorities mandate Good Manufacturing Practice (GMP) compliance, requiring manufacturers to validate each batch’s purity, potency, and stability through advanced analytical techniques and process controls that can be costly and technically challenging [86, 87]. Additionally, many miRNA-based drugs incorporate chemical modifications (e.g., 2’-O-methyl, phosphorothioate linkages) to enhance stability and reduce immunogenicity, which must be rigorously tested to ensure they do not alter the therapeutic efficacy or introduce unintended toxicities [88].

From a regulatory perspective, the approval pathway for miRNA-based therapies follows the evolving framework established for oligonucleotide-based drugs. Guidelines such as ICH M3(R2) outline the necessary toxicology, biodistribution, and long-term safety assessments, required before clinical trials can commence. Additionally, companion diagnostics (CDx) may be required if miRNA therapies are designed for biomarker-driven patient stratification, further adding to the regulatory burden. The FDA’s Breakthrough Therapy Designation or EMA’s PRIME (Priority Medicines) program may offer accelerated pathways for promising miRNA therapies, but meeting these criteria requires substantial early-stage clinical evidence.

From a commercial perspective, the high costs associated with the development and production of miRNA therapies, compounded by the stringent regulatory requirements, may deter investment and delay market entry. Pharmaceutical companies must allocate relevant resourced to developing precise and scalable delivery platforms. miRNA drugs need to be thoroughly evaluated for their cost-effectiveness. This involves assessing the overall cost of development (including research, clinical trials, regulatory approvals, and manufacturing related to the potential value in terms of health benefits. For miRNA-based therapies an additional challenge can be due to overlapping patents on miRNA sequences, chemical modifications, and delivery technologies, potentially limiting commercial viability [89, 90].

A comprehensive analysis of the potential market size for miRNA-based therapies is critical. This includes a clear assessment of the number of patients who could benefit from miRNA drugs in different diseases (such as cancer, cardiovascular diseases, or neurological disorders). Additionally, the need of addressing global therapy needs should be considered, considering regional differences in healthcare infrastructure and accessibility to advanced treatments with special refer to low-medium income countries. On the other hand, a larger target population, coupled with the ability to treat multiple diseases, enhance the commercial potential of miRNA-based therapies.

Moreover, pricing for miRNA drugs must reflect the treatment’s novelty, potential benefits, and costs. However, it is also essential to consider how prices are set in the context of competition with existing therapies. If the price of miRNA drugs is set too high, it may limit their accessibility, particularly in markets with reduced healthcare resources. On the other hand, pricing it too low could undercut the revenue potential needed to cover research and development costs. A balanced approach to pricing, considering the payer landscape, reimbursement strategies, and economic constraints in different markets, is essential for the commercial success of miRNA drugs.

Despite these challenges, advances in artificial intelligence-driven drug discovery, high-throughput screening and next-generation delivery technologies are helping to accelerate the clinical translation of miRNA therapeutics. With regulatory frameworks evolving to accommodate RNA-based drugs, addressing safety, manufacturing and commercial scalability will be key to unlocking the full therapeutic potential of miRNA-based interventions.

However, the entire field of RNA therapeutics exhibits greater plasticity compared to other approaches. This was demonstrated during the COVID-19 pandemic by the rapid availability of vaccines just months after virus was sequenced, as well as the swift adaptation to next- generation, mutation-driven vaccines. The same applies to miRNA therapeutics, which benefit from enhanced modelling through artificial intelligence prediction tools.

In conclusion, while miRNA therapies hold high potential, the path to regulatory approval and commercial success is fraught with challenges. Ensuring consistent quality, addressing safety concerns and developing effective, scalable manufacturing processes are essential to overcoming regulatory hurdles. These regulatory and commercial challenges underscore the need for ongoing research and innovation to optimize miRNA therapeutic development, enhance delivery strategies, and reduce manufacturing costs to lead to market successfully these promising treatments [7, 91].

### Current clinical trials and outcomes on MiRNA therapeutics in cancer treatments

miRNA-based therapies in clinical trials can be broadly categorized into two strategies: miRNAs as drugs which substantially refer to miRNA mimics to restore tumor-suppressive miRNA expression, and miRNAs as targets, which focus on miRNA inhibitors or a variety of inhibitory approaches, including ASOs, LNAs or miRNA sponges, to silence oncogenic miRNAs. Table 3 list the most relevant recent clinical trial in the treatment of solid and hematological malignancies.

**Table 3:** Most relevant recent clinical trial in the treatment of solid and hematological malignancies

Target miRNA	Drug	Molecular Type	Modification & Delivery	Cancer target	Stage	ID Status	Route of Administration	Sponsor/ Company	Reference
miR-34a	MRX34	Mimic	Liposome-formulated (NOV40)	Liver cancer, NSCLC, renal cell carcinoma, lymphoma, melanoma, advanced solid tumors	Phase I/Ib	NCT01829971 Completed NCT02862145Terminated	Intravenous	Mirna Therapeutics	[92], [93]
miR-16	TARGOmiRs	Mimic	Bacterial minicell-derived EVs	Malignant pleural mesothelioma, NSCLC	Phase I	NCT02369198 Completed	Intravenous	Asbestos Diseases Research Foundation	[94]
miR-193a	INT-1B3	Mimic	Lipid nanoparticle	Advanced Solid Tumors	Phase I/Ib	NCT04675996Terminated	Intravenous	InteRNA	
miR-221	LNA-i-miR-221	Antisense oligonucleotide (ASO)	LNA-modified	Advanced solid tumors, multiple myeloma, refractory hepatocarcinoma	Phase I	NCT04811898Completed	Intravenous	Azienda Ospedaliera Universitaria Mater Domini, Catanzaro, Italy	[53]
miR-155	Cobomarsen	Antisense oligonucleotide (ASO)	PS backbone modification	Cutaneous T-cell lymphoma (Mycosis Fungoides Subtype), DLBCL	Phase I/ II	NCT02580552Completed NCT03713320Terminated	Intravenous	miRagen Therapeutics, Inc	
miR-10b	TTX-MC138	AMO	Dextran-coated iron oxide nanoparticle	Advanced solid tumors	Phase 0 Phase I/II	NCT05908773Completed NCT06260774Recruiting	Intravenous	TransCode Therapeutics	
miR-21	Lademirsen	Chemically modified ASO	PS-modified backbone	Alport syndrome, renal disfunction	Phase II	NCT02855268Terminated	Subcutaneous	Genzyme, a Sanofi Company	[99]

One of the most prominent examples of miRNAs as drugs is MRX34, a liposome-formulated mimic of miR-34a, a well-characterized tumor suppressor. Notably, miR-34a was the first miRNA mimic tested in cancer patients, marking a major milestone in the clinical translation of miRNA therapeutics. By restoring miR-34a expression, MRX34 aimed to suppress oncogenic pathways and induce apoptosis. MRX34 was evaluated in a standard 3 + 3 dose escalation Phase I clinical trial (NCT01829971), given intravenously twice weekly (BIW) for three weeks in 4-week cycles, with oral dexamethasone premedication, to assess the maximum tolerated dose (MTD), safety, PKs, and clinical activity. Results in forty-seven patients with various solid tumors including liver cancer, non-small cell lung cancer (NSCLC), renal cell carcinoma, melanoma, lymphoma, and other advanced solid tumors indicated acceptable safety and showed evidence of antitumor activity in a subset of patients with refractory advanced solid tumors. The MTD for the BIW schedule was 110 mg/m2 for non-HCC and 93 mg/m2 for HCC patients84 defined as recommended Phase 2 dose (RP2D) [92]. In an expansion cohorts consisting in 85 adults with various solid tumors refractory to standard treatments, MRX34 used at the RP2D was observed a manageable toxicity profile in most patients and some clinical activity. A multicenter Phase 1B study (NCT02862145) included 3 cycles of MRX34 treatment given over an approximately 8 week period was designed to investigate the biomarkers, PKs and PDs in patients with melanoma patients with easily accessible lesions. Unfortunately, the trial was terminated due to severe immune-related adverse events (AEs), that resulted in four patient deaths, underscoring safety and delivery challenges in the miRNA mimic therapy [93].

Another innovative approach involving miRNA mimics is TARGOmiRs, a bacterial minicell-derived extracellular vesicle delivery system carrying a mimic of miR-16. This therapy was evaluated in a traditional 3 + 3 dose-escalation Phase I trial (NCT02369198) for malignant pleural mesothelioma and NSCLC, treated via 20 min intravenous infusion (IV) either once or twice a week (3 days apart) at a dose of 5 × 10^9^ per week, in 27 patients included in five dose cohorts. By reintroducing miR-16, a key tumor suppressor, TARGOmiRs demonstrated acceptable safety profile and early sign of tumor-suppressive effects with one patient (5%) achieving a partial response and 15 (68%) had stable disease, warrants further clinical investigation in larger studies [94].

A lipid-nanoparticle formulated with a double stranded chemically-modified miR-193a-3p mimic is also under investigation in a Phase I/Ib trial (NCT04675996) based on promising preclinical findings [95, 96]. miR-193b acts as a tumor suppressor, and its transient restoration through lipid nanoparticle (LNP), formulated with chemically modified miR-193b (INT-1B3) mimic treatments has strong anti-leukemic effects in a clinically relevant system based on PDXs triggering a long-term T cell mediated immune response against tumor antigens via the induction of immunogenic cell death and modulation of the tumor microenvironment. INT-1B3 clinical investigations were performed in a two-phase dose escalation first-in-human study, to evaluate the safety, PKs, PDs, and preliminary efficacy in the treatment of patients with advanced solid tumors. Both in phase 1 and phase 1b patients were treated with 60-min IV infusions twice per week in 21-day cycles. Unfortunately, the study was terminated for insufficient funding and to our knowledge, no clinical outcome has been reported.

On the other hand, therapies targeting oncogenic miRNAs (miRNAs as target) have shown considerable therapeutic promise. A landmark example is the recently completed study on LNA-i-miR-221, the very first LNA miRNA-inhibitor evaluated in cancer patients with refractory advanced disease. In this phase I, dose-escalation trial (NCT04811898), LNA-i-miR-221 demonstrated an excellent safety profile and promising clinical activity. Patients with progressive cancers received IV infusions of LNA-i-miR-221. No grade 3–4 toxicities or dose-limiting toxicities was observed, and the MTD was not reached. Notably, 50% of patients achieved disease control (stable disease), and one patient with colorectal cancer experienced a durable partial response lasting over three years and presently is still alive. PD analyses confirmed the downregulation of miR-221 and the upregulation of its canonical targets, CDKN1B/p27 and PTEN. These encouraging findings support further the clinical development of LNA-i-miR-221 as a promising novel therapeutic agent to be investigated in a phase II study for solid tumors [53].

Another, LNA-ASO is Cobomarsen (MRG-106), targeting miR-155, a miRNA highly implicated in hematological malignancies [97]. Cobomarsen is being investigated in Phase I and II trials (NCT02580552; NCT03713320) for cutaneous T-cell lymphoma (CTCL) and diffuse large B-cell lymphoma (DLBCL). Using chemically stabilized ASOs with phosphorothioate (PS) backbones, Cobomarsen aims to inhibit miR-155, thereby restoring normal gene expression and reducing tumor progression [97, 98]. Phase I was successfully completed, demonstrating favorable and safety profile across 45 patients treated with 75 mg intra tumoral or 300, 600, 900 mg IV dose and improvements in disease and quality of life over the first 100 days on study drug. Phase II study (SOLAR) was then expanded to include patients with CLL, DLBCL, and ATLL diseases, characterized by increased miR-155 expression. Patients received 300 or 600 mg infusions that showed similar efficacy and tolerability, offering the most consistent response rate based on skin mSWAT scores in Phase 1 study. However, it was ultimately terminated for changes in firm strategies rather than for safety concerns or lack of efficacy.

The investigational agent TTX-MC138 represents another miRNA-targeted approach. TTX-MC138, LNA-based antagomirs, TransCode’s proprietary, was delivered to metastatic sites by aminated dextran-coated iron oxide nanoparticles, to specifically inhibits miR-10b, a key miRNA driving tumor metastasis. In the Phase 0 microdose trial (NCT05908773), in subjects with advanced solid tumors and radiographically confirmed metastases, was assessed the PK and biodistribution of TTX-MC138 as determined by PET-MRI. The phase I/II multicenter, open-label, dose-escalation and expansion study of TTX-MC138 (NCT06260774) is now recruiting patients with advanced solid tumors. Three study periods are designed: Screening (up to 28 days), treatment is based on 28-day cycles, with dosing on Day 1, and survival follow- up. Each 28-day cycle includes 1 dose of study drug administered as an IV on Day 1, starting from 0.4 mg/kg to 3.2 mg/kg or MTD assessment. TTX-MC138 aims to suppress metastatic progression, offering a novel therapeutic option for patients with highly invasive cancers.

Additionally, Lademirsen, an ASO targeting miR-21, is being evaluated in a Phase II trial with a randomized, double-blind, placebo-controlled period followed by an open-label period (NCT02855268). The objective of the study was to investigate the effect of miR-21 inhibition on rate of eGFR decline in adults with Alport syndrome (AS) at risk of rapid disease progression. MiR-21 is an established oncomiR overexpressed in various cancers, contributing to tumor proliferation and chemoresistance. By silencing miR-21, Lademirsen seeks to reverse oncogenic processes and restore tumor suppressor gene function. Preclinical models of AS disease have suggested that targeting miR-21 may slow the decline in kidney function in individuals affected by the disease. Forty-three adults with AS participated and 28 completed 48 weeks of double-blind treatment. No significant differences between groups were identified in eGFR at any timepoint. The study results summarize that while anti-miR-21 therapy with Lademirsen was generally well-tolerated with an acceptable safety profile, no meaningful improvement in rate of kidney function decline in adults with AS at risk of rapidly progressive disease was however observed [99].

Collectively, these clinical trials demonstrate a growing interest for the therapeutic potential of miRNA-based strategies, including the use of miRNA mimics to restore tumor suppressors or miRNA inhibitors to silence oncogenic miRNAs. Advances in delivery systems, such as lipid nanoparticles, extracellular vesicles, and nanoparticle-based carriers, alongside chemically stabilized oligonucleotides like LNAs are driving the clinical success of these treatments. Despite challenges related to delivery optimization and immune responses, ongoing trials underscore the feasibility of miRNA-based therapies and their potential to transform cancer treatment.

An outstanding point is the effect of miRNA therapeutics on patient quality of life. Although this aspect can be formally addressed only on large patient cohorts by assessing Patient Reported Outcomes (PROs), the first-in-human study by Tassone et al. provided preliminary evidence of an improvement of neurotoxicity symptoms in late-stage cancer patients treated with LNA-i-miR-221. This finding may have a solid rationale in the pleiotropic effects of miR-221 on neuronal conduction (unpublished results).

### Future directions and conclusion

The progression of miRNA-based therapeutics from preclinical studies to clinical trials marks a significant advancement in precision oncology. Despite challenges such as delivery specificity and potential toxicity, ongoing innovations are enhancing miRNA stability and refining targeted delivery systems to overcome these obstacles.

An exciting future direction lies in the potential for combining miRNA-based therapies with existing treatment modalities, such as chemotherapy, immunotherapy, or targeted therapies. These synergistic approaches may enhance therapeutic efficacy by leveraging miRNA’s ability to regulate multiple oncogenic pathways and improve tumor sensitivity to conventional treatments. Additionally, miRNA-based therapies could play a pivotal role in addressing drug resistance and treating metastatic cancers, where current treatment options remain limited.

It must be considered that miRNAs can play a regulatory role which might be context dependent. While the expression of the miR-221/222 can have a favourable prognostic role at diagnosis as recently demonstrated by Soureas et al. [100], it appears to become an highly remarkable therapeutic target in refractory disease where its inhibition may lead to tumor growth control by inducing senescence and dormancy, which might be detrimental in drug sensitive disease under active treatment. This concept may lead to a paradigm change in tumor treatment based on longitudinal monitoring of the disease by single cell genomics or epigenomic signatures in the circulating tumor fractions offering the opportunity to select treatment timing.

A challenging issue is the use of novel study designs for early trials including biomodulatory effects as co-primary endpoints in seamless platform developmental paths which may derive major benefit from Artificial Intelligence. Another and correlated transformative aspect is the use of miRNA expression profiles for tailoring treatments to individual patients. By identifying unique predictive miRNA signatures, clinicians could better stratify patients, and design personalized therapeutic regimens, thereby advancing the goals of precision medicine.

Future research will likely focus on optimizing delivery systems, minimizing off-target effects, and expanding the clinical applications of miRNA therapies. Furthermore, studies exploring the combination of miRNA modulation with immune checkpoint inhibitors or CAR-T cell therapies could unlock new avenues for immuno-oncology.

Ultimately, miRNA therapeutics hold the promise of revolutionizing cancer care, offering a multifaceted approach to target both oncogenic drivers and tumor-supportive microenvironments. With continued advancements, miRNA-based treatments may emerge as integral components of next-generation oncology protocols, providing innovative solutions for improving patient outcomes in resistant advanced cancers.

## Data Availability

No datasets were generated or analysed during the current study.
